# Smoking and Cervical Cancer

**DOI:** 10.5402/2011/847684

**Published:** 2011-07-14

**Authors:** José Alberto Fonseca-Moutinho

**Affiliations:** Faculdade de Ciências da Saúde, Universidade da Beira Interior, Avenida Infante D. Henrique, 6200-506 Covilhã, Portugal

## Abstract

Cervical cancer (CC) is the third most common cancer in women worldwide; however, CC is a preventable disease, and much effort should be done to prevent it. Persistence of high-risk HPV infection is the strongest epidemiologic risk factor for CC, however it is not sufficient for development of the disease it cofactors should be present. In 2004; IARC listed cervical cancer among those causally related to smoking. Smoking interferes with incidence and prevalence of HPV infection and is associated with cervical intraepithelial neoplasia and invasive CC. Multiple factors seem to intervene on cervical carcinogenesis related with tobacco, especially by direct local carcinogenic effect and local immunosuppression. Smoking addition is also closely related with other confounding factors, like unfavorable psychosocial events, systemic immunity, contraception, and nutrition, which got difficult epidemiologic evaluation of smoking role on cervical carcinogenesis. Smoking habits should be taken in account in clinical practice and in research concerning CC.

## 1. Introduction

Cervical cancer (CC) is the third most common cancer in women, and the seventh overall, with an estimated 530 000 new cases in 2008. More than 85% of the global burden occurs in developing countries, where it accounts for 13% of all female cancers. Cervical cancer is responsible for 275 000 deaths in 2008, about 88% of which occur in developing countries: 53 000 in Africa, 31 700 in Latin America and the Caribbean, and 159 800 in Asia [1]. However, CC is preventable disease, and much effort should be done to prevent it. 

It is now well-established that persistence of Human Papillomavirus (HPV) infection is the strongest epidemiologic factor associated with intraepithelial neoplasia and cancer of cervix [2–5], and it is considered a necessary cause for development of the disease but not sufficient. Exogenous or endogenous factors had been identified to, in conjunction with HPV infection, influence the risk for CC. As Castellsaguè and Muñoz [6] suggested, cofactors may be classified into three groups: (1) environmental or exogenous cofactors, including use of oral contraceptives (OCs), tobacco smoking, diet, cervical trauma, coinfection with human immunodeficiency virus (HIV) and other sexually transmitted agents; (2) viral cofactors, such as infection by specific types, coinfection with other types, HPV variants, viral load, and viral integration; (3) host cofactors, including endogenous hormones, genetic factors such as human leukocyte antigen and other host factors related to the host's immune response. 

Winkelstein Jr. [7] in 1977 was the first to put the hypothesis that smoking is a risk factor for cervical cancer. Since then, the action of tobacco on cervical carcinogenesis has been a matter of scientific debate. 

The objective of this paper is to update the state of the science about smoking and cancer of the cervix.

## 2. Material and Methods

It was checked up in PubMed/Medline and Embase databases, studies written in English with keywords: cervical cancer; cervix; CIN; HPV; tobacco; smoking and smoke. Only meta-analysis and or multi-institutional studies were considered for analysis.

## 3. Results

Plummer et al. reported the first multicentric case-control study in 2003 [8]. In analyzing eight studies on invasive cancer and two on carcinoma *in situ*, conducted by International Agency for Research on Cancer (IARC), between 1985 and 1997, the authors conclude that ever-smokers have an excess risk of cervical cancer that persists after controlling for the strong effect of HPV and for other potential cofactors of progression from infection to cancer, and they suggest that squamous cell carcinoma of the cervix should be added to the list of tobacco associated cancers, while for adenocarcinoma, further data should be warranted. 

In 2004, IARC revisited its previous conclusions and listed cervical cancer among those causally related to smoking [9]. 

In 2006, a collaborative reanalysis of 12 studies on CC [10], enrolling 8,097 cases of squamous cell carcinoma, 1,374 women adenocarcinoma, and 26,445 women without carcinoma of the cervix, showed that there are no substantial differences between the two most common histological types of invasive cervical cancer with respect to the role of number of sexual partners, age at first intercourse, age at first birth, body mass index, and use of oral contraceptives. The exception was current tobacco smoking, which is associated with an increased risk of squamous cell but not for adenocarcinoma of the cervix, in agreement with other studies [11, 12]. 

Vaccarella et al. [13] in 2008 reported a pooled analysis of 13 IARC HPV prevalence survey in 11 countries worldwide, carried out between 1993 and 2005. They concluded that current tobacco smoking was associated with a significant, although moderate, increased risk of prevalent HPV infection. Among current smokers, the risk of being HPV-positive increased with increasing number of cigarettes smoked per day, and women who reported to smoke 15 or more cigarettes daily had a 2-fold risk of HPV positivity as compared with never-smokers. These results have shown that smoking habits interfere with prevalence of HPV infection in agreement with reported in previous studies [14–16]. 

Syrjänen et al. [17], in 2007, in analyzing a cohort of 3,187 women, conclude that cigarette smoking was not an independent risk factor of CIN2+, except for those patients who tested for HR-HPV (high-risk HPV), and current smoking remains an independent predictor for those patients in a multivariate model, in agreement with previous study of Harris et al. in 2004, [18] that found among women with oncogenic HPV infection, smoking was associated with risk for both CIN1 and CIN2-3. Of the three smoking measures (smoking status, pack years of exposure, and number of cigarettes per day), number of cigarettes per day (>10 cigarettes) was the most strongly associated with risk for CIN1 and CIN2-3. In this study, association between cigarettes smoked by day and CIN did not appear to be mediated by an immunologic response. Those studies suggest that smoking has a special adverse effect on HR-HPV driven cervical carcinogenesis. 

A recent study [19], published in 2010, conducted on 2,011 women, 15–19 years old, recruited from 1988 to 1992 then regularly followed until 1997, concluded that there is no evidence to suggest that the risk of acquiring a HPV infection of any type, or a HPV16 or HPV18 infection, increases with either pack years of exposure to smoking or duration of current smoking episode, suggesting that smoking is not a important risk factor for HR-HPV infection.

Xi et al. in 2009 [20] reported an analysis of 1,050 women HPV16 and/or HPV18 positives for viral DNA load, enrolled into the ASCUS-LSIL Triage Study. The authors concluded that higher HPV16 and HPV18 DNA load was associated with status of current, but not former, smoker. Among current smokers, the viral load did not appear to vary appreciably by the intensity and duration of cigarette smoking, in accordance, with previous study of Gunnell et al. [21] in 2006, that in testing for HPV16 DNA presence in first archival cervical smears from 375 cases of *in situ* cervical squamous carcinoma (CIS) and in 363 controls, it was found that current smokers with a high HPV16 viral load at time of first smear were at a particularly increased risk (27-fold) compared with current smokers without HPV-infection.

## 4. Discussion

Some molecular mechanisms have been suggested through why smoking may contribute towards cervical carcinogenesis: one involves direct exposure of the deoxyribonucleic acid (DNA) in cervical epithelial cells to nicotine and cotinine, and the other involves exposure to metabolic products resulting from the reactions of other components of cigarettes such as aromatic polycyclic hydrocarbons and aromatic amines. [22, 23]. Cervical mucus of smokers contains measurable amounts of cigarette constituents and their metabolites such as benzo[*a*]pyrene (BaP) [24], nicotine, and nicotine derived nitrosamines 4-(methylnitrosamino)-1-(3-pyridyl)-1-butanone [25]. BaP up regulation of HPV genome amplification may increase the probability of viral DNA integration into the host genome, a milestone in the development of cervical cancer [26]. The *in vivo* effects of long-term nicotine exposure could affect persistent cellular proliferation, inhibition of apoptosis, and stimulation of vascular endothelial growth factor, with increased microvessel density [27]. Other mechanisms that may explain smoking-related carcinogenesis are related with abnormalities in the systemic and peripheral immune systems of smokers, that include unbalanced systemic production of pro- and anti-inflammatory cytokines [28], elevated numbers of cytotoxic/suppressor T lymphocytes, suppression of T lymphocyte activity, diminished numbers of helper T lymphocytes, decreased numbers of natural killer lymphocytes and low levels of immunoglobulins other than immunoglobulin E (IgE) [29]. These effects may result from substantial decreased numbers of Langerhans cells in the cervix of smokers [30, 31]. Aberrant HPV-induced DNA methylation may be another mechanism to explain cervical smoking-related carcinogenesis. *In vitro* studies in untransformed and transformed cell lines show that short-term exposure to nicotine or cigarette smoke extract is followed by changes in the expression of the DNA methyltransferases DNMT1, DNMT3A,and DNMT3B. Aberrant methylation of the tumour suppressor gene, p16 (CDNK2A), is strongly associated with current smoking in women with squamous cell cervical cancers and high-grade CIN [32]. 

Intriguing studies analyzing wood-smoke effect on cervical carcinogenesis have shown that, among HPV-positive women, a dose-response relationship is observed for exposure to wood smoke and cervical cancer that persisted in multivariate analysis [33], and chronic exposure to wood smoke significantly increased the risk of CIN III, suggesting that chronic inhalation of carcinogens derived from wood smoke could have an effect on the progression to cervical cancer, similar to that observed for cigarette smoking [34]. Probably the smoking effect on cervical carcinogenesis is not tobacco-specific.

Smoking seems to affect negatively the early natural history of HPV infections. The regression of Low Squamous Intraepithelial Lesions (LSIL) within 2 years is significantly lower in smokers than in never-smokers [35]. 

Smoking affecting clearance HPV infection remains a conflicting issue. For some authors, smoking have no influence in duration of HPV infection [19], for others tobacco delays the clearance of HPV infection [36, 37].

Covalent alteration of DNA to form DNA adducts is considered an early step in chemical carcinogenesis and, therefore, detection of DNA adducts provides evidence of exposure of the cervix to carcinogens. Prokopczyk et al. [38] had shown, in accordnce with previous studies, that significantly higher DNA adduct levels are present in the cervix of smokers as compared with nonsmokers, providing molecular evidence of smoking-related carcinogenic agents that affects the DNA of the cervical epithelium. However, the authors reported no significant differences in smoking-related DNA damage (DNA adduct levels) between HPV-positive and HPV-negative smokers, suggesting that smoking DNA damage is not related with HPV infectivity.

Genetic susceptibility to smoking is an important issue. Cervical cancer risk in smokers may be modified by genetic variants, as that described to interleukin 2 [39] or to 8q24 chromosome polymorphisms [40]. In a recent study [41], the tumor suppressors p53, the fragile histidine triad and the interleukin-10 were under-expressed, and the cyclooxygenase-2 and the Ki-67 were over-expressed in smoking, compared with nonsmoking women with CIN.

Familial and social factors are determinant for smoking attitudes. Being older, being divorced, having friends/family who smoke, and having parents who smoke are all associated with ever smoking, and friends are the primary factor influencing ever smoking, especially among younger women [42]. Smokers seem to have lower compliance for cervical cancer screening, the most powerful weapon against cervical cancer mortality. Smokers held less positive attitudes towards cervical screening than did nonsmokers [43], and the level of nicotine dependence is also significantly related to compliance with screening recommendations; women with higher levels of dependence are less likely to be compliant [44]. 

Unhealthy life style, negatively rated life events, lack of social support, coping style, and distress, often associated with smoking, alcohol abuse, and illegal drugs addition, have been reported as risk factors for cervical cancer [45–47], especially among low-educated women [48, 49]. 

Hormonal contraception is implicated on cervical cancer development. Appleby et al. [50], in analyzing 24 studies worldwide that included 16,573 women with cervical cancer and 35,509 without cervical cancer, concluded that the relative risk of cervical cancer is increased in current users of oral contraceptives, and that increased risk is higher in current smokers women. 

Nutrition is another important factor that seems to influence cervical carcinogenesis. Oxidative stress induced by deficiencies in antioxidant micronutrients is likely to change normal redox balance and transform the HPV-infected cells toward a carcinogenic process in the cervix [51]. Folate is a well-studied micronutrient with effect on cervical carcinogenesis. Reduced immunocompetence associated with deficiencies of folate and vitamin B12 could increase the risk of infection and persistence with multiple types or higher viral loads of HR-HPVs [52–54]. 

Higher prevalence rates of HPV have also been found in HIV-seropositive women, proportional to their level of immunosuppression [55–57]. HIV-seropositive women, and particularly the current smokers, are at significantly elevated risk for the development and recurrence of precancerous and cancerous cervical disease [58, 59], especially those who have lower levels of TH cells. It has been shown that TH cells are involved in the defense against HPV-transformed cells [60]. Hence, immunosuppression in general, and depletion of TH cells in particular, may interact with HPV infection to increase the risk of cervical cancer. Marijuana usage is another described factor that potencies tobacco immunosuppression [61]. 

Smoking also affects survival among women diagnosed with cervical cancer. Recently, Coker et al. [62], in analysing 2661 women diagnosed with invasive cervical cancer from 1995–2005, found that, after adjustment for age and stage at diagnosis, cell type, rural residence, race, insurance coverage, and treatment-received, current smoker, were 35% more likely to die of any cause and 21% more likely to die of cervical cancer compared with known nonsmoking cases, in accordance with previous studies. Unfortunately, few smokers with cervical cancer quit or decreased consumption during treatment [63].

## 5. Conclusions

All meta-analyses and multi-institutional studies point out that smoking is an important cofactor for cervical squamous cancer and probably also for cervical adenocarcinoma.

Acquisition of HR-HPV infection seems to be a smoking independent event; however, progression of the acquired infection is negatively affected by current smoking. Former smoking seems to be no so important.

The action mechanisms of smoking on HPV driven cervical carcinogenesis seem to be complex and multifactorial. In current smokers, genetic, immunologic, and dietary factors may be associated with cervical epithelium susceptibility for HPV carcinogenic effect, but womens unhealthy lifestyles seem to be major factors associated with cervical carcinogenesis. What is much determinant for cervical cancer susceptibility: tobacco action by its own or women health or behaviour associated with smoking habits? Until now, a scientific-based answer is lacking. Epidemiologic, clinical, and laboratorial research is needed to elucidate this unsolved topic. 

In order to prevent cervical cancer and to improve therapy results, the practitioner should understand negative effects of tobacco on cervical carcinogenesis, and in those current smoking women with diagnosis of cervical HPV infection or CIN, and he must have an active intervention to invite them to quit smoking habits.

The researchers on cervical cancer should be aware that smoking habits are a major cofactor on cervical HPV driven carcinogenesis, and an important confounder factor for research. Smoking effects should be taken in account by all researchers in their studies.
